# Consideration of high-quality development strategies for soil and water conservation on the loess plateau

**DOI:** 10.1038/s41598-022-12006-w

**Published:** 2022-05-18

**Authors:** Jinliang Zhang, Yonggang Ge, Gaoang Yuan, Zhiyu Song

**Affiliations:** 1 Yellow River Engineering Consulting Co. Ltd, Zhengzhou, 450003 China; 2 Key Laboratory of Water Management and Water Security for Yellow River Basin of Ministry of Water Resources (Under Construction), Zhengzhou, 450003 China; 3 Zhengzhou University, Zhengzhou, 450003 China

**Keywords:** Civil engineering, Sustainability, Environmental impact

## Abstract

The construction of check dams is an important measure to prevent soil erosion on the Loess Plateau and reduce the amount of sediment entering the Yellow River. Based on an analysis of the current situation of soil and water conservation on the Loess Plateau and the three major problems faced by the traditional homogeneous soil check dam construction, the study of anti-scouring materials, hydrological calculation methods, dam design and construction technology and soil and water conservation monitoring are carried out in this paper. The results showed that the current soil and water conservation measures on the Loess Plateau have achieved remarkable outcomes. The new design and application concept of check dams with anti-burst and multi-sand interceptions is innovatively proposed in this paper. The new materials of solidified loess have good durability and anti-scouring characteristics and could meet the overflow and anti-scouring requirements of the new check dam. The small watershed high sand content of hydrological calculation can establish the upper limit of the flood sediment boundary for the anti-scouring protection layer of the check dam. The new technology of dam design and construction can achieve no collapse or slow collapse when encountering floods exceeding the standard. Intelligent monitoring systems can realize real-time dynamic monitoring for soil and water conservation on the Loess Plateau. The results will eventually contribute to the national strategy of the Ecological Protection and High Quality Development in the Yellow River basin.

## Introduction

The Loess Plateau area is a typical ecologically fragile area in China and has the most serious soil erosion in the world^1,2^. The accumulation of large amounts of sediment loss makes the riverbed in the lower reaches of the Yellow River higher than the ground, which seriously threatens flood control measures and ecological security in northern China^3–5^. The government has attached great importance to soil and water conservation in the Loess Plateau area and has carried out large-scale scientific research, obtaining world-renowned achievements^6–8^. The implementation of major national strategies for Ecological Protection and High Quality Development in the Yellow River basin has brought a new historical opportunity for the development of soil and water conservation in the Yellow River basin. Vigorously promoting Loess Plateau soil and water conservation and strengthening ecological protection are fundamental measures to protect the long term stability of the Yellow River and promote the quality development of the Yellow River basin^9–11^. Higher requirements for soil and water conservation monitoring have been proposed for the Loess Plateau.

Soil erosion in the Loess Plateau area has three main characteristics. First, the area of soil erosion is widespread. Within the entire Loess Plateau, which has an area of 640,000 km^2^, approximately 460,000 km^2^ of soil experiences different degrees of soil erosion. Second, the intensity of soil erosion is high. The Yellow River delivers 1.6 billion tons of sand downstream every year. The sediment of the Yellow River is deposited when the downstream speed slows; thus, these deposits accumulate year after year, forming above-ground rivers and a relatively large extension of land at the mouth of the sea. Then, the rate of soil erosion is fast. The average thickness of soil erosion on the Loess Plateau is 1 cm every year, and such serious soil erosion is caused by both natural and human factors^12–14^. Due to population growth, when survival became the primary issue, people deforested the land and increased the area of arable land while weakening its natural buffering capacity, leading to a break in the stable state of the otherwise fragile ecosystem in some areas and resulting in serious soil erosion^15^.

Preventing soil erosion in the Loess Plateau region can reduce the amount of sediment entering the Yellow River, improve the ecological environment, reduce the frequency of natural disasters, promote industrial restructuring, and increase people's economic income^16^. A sectional measurement was adopted to improve the soil and water conservation on the Loess Plateau in the past. First, engineering measures, such as check dams, are used to reduce soil erosion, enhance the control of soil loss, and reverse siltation. Second, biological measures are used to drain and conserve the soil, and under good circumstances, vegetation can be restored. The last utilized aspect includes vegetation measures, which are a reasonable development and use according to the topography and other characteristics and are used as a buffer or protection land in an area that cannot be developed^17–20^. This has attracted the attention of scholars, and in-depth research has been conducted, with many beneficial conclusions obtained, providing a scientific basis for the ecological research on the Loess Plateau^21,22^. Soil and water conservation plays a significant role in studying the hydro-biogeochemical and water-carbon coupling cycle of the Loess Plateau^23^.

**Figure 1:**
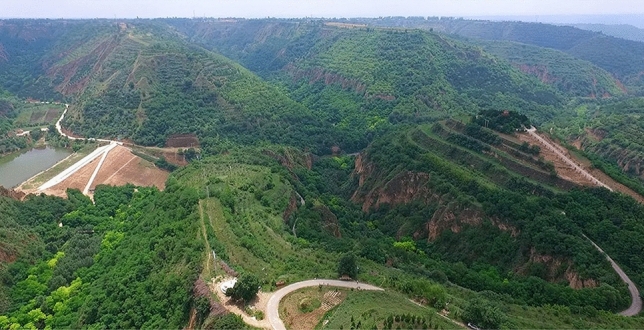
Effects of soil and water conservation on the Loess Plateau.

Although soil erosion on the Loess Plateau is still relatively serious, after years of treatment, it has been greatly improved. The Nanxiaohe Demonstration Area in Qingyang City, Gansu Province, is taken as an example, and it is shown in Fig. 1. In recent years, the main trends in soil and water conservation on the Loess Plateau area have been as follows: the amount of sand transported by the Yellow River has decreased to a historically low level; the vegetation cover of the Loess Plateau has increased dramatically; the restoration of vegetation on the Loess Plateau is approaching the limit of sustainable use of water resources; and the contribution of human activities has reached an unprecedented level. Soil and water conservation on the Loess Plateau should receive more attention, and the concept of water conservation in the new era should be more refined. Soil and water conservation not only reduces soil erosion and increases arable land area but also, more importantly, enhances landscape quality, improves the human living environment, optimizes economic and industrial structures, and boosts regional social and economic growth^7,24^. Guided by the concept of considering mountains, water, forest, fields, lakes, grass, and sand as a living homogeneous body, practising the green development concept in which green water and green mountains are golden mountains, building a new model of soil and water conservation through the deep coupling of regional soil and water conservation and socioeconomic development, enhancing the sustainable development of regional society and economy, and aiding in the formation and construction of a stable mechanism for poverty eradication are of important economic and social benefit.

## Materials and methods

### Data collection and analysis

**Figure 2:**
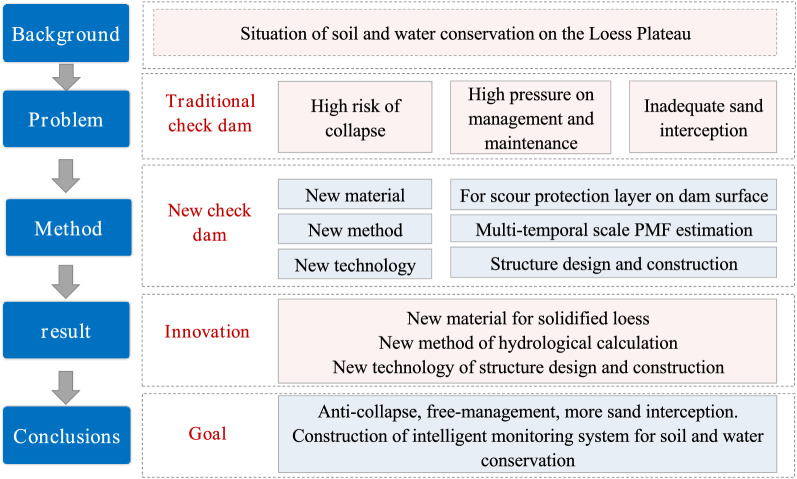
The research idea and technical route of this research.

Information on the status of soil erosion and conservation management on the Loess Plateau is obtained from the available literature. According to the data analysis and laboratory test conclusions, the research ideas and technical route of this paper are shown in Fig. 2. Based on the analysis of the current situation of soil and water conservation on the Loess Plateau, the importance of check dam construction is analyzed, and the three major technical difficulties faced by traditional silt dams are discussed. Then, a new theoretical and technical system for check dams is proposed, which realizes a new method of PMF estimation in an extra-small watershed, a new structure of dam construction, new materials for solidified loess, and new construction technology. The economic and technical benefits of traditional check dams and new check dams are compared and analyzed to provide a scientific basis for the popularization and application of new check dams. Finally, the intelligent monitoring system for soil and water conservation on the Loess Plateau is constructed based on information technology.

### Sampling and testing

The loess was obtained from a slope in Qingyang city, Gansu, China. The soil stabilizer was a new product that was developed independently, and it is one kind of gray powdery hydraulic cementing material. The soil stabilizer is composed of slag, fly ash, gypsum, composite activator and surfactant and other materials. It is a cement fly ash type stabilization material. Its chemical composition is 51.25% CaO and 30.36% SiO_2_, and it also contains a small amount of Fe_2_O_3_, Al_2_O_3_, MgO, etc. The fineness (80 µm) is 25.6%, the initial setting time is 134 min, and the relative density is 2.0. Tap water was used in the experiments.

A specimen with a size of Φ50 mm × H50 mm was adopted in this study. Unconfined compressive strength (UCS), water absorption rate, anti-scouring performance and freeze–thaw cycle tests were carried out to discuss the performance of the new material for solidified loess by the microcomputer-controlled electronic universal testing machine. Note that four samples were tested for strength and the average values were adopted.

## Results and analysis

### The problems encountered by traditional check dams

Check dams are an effective measure for soil and water conservation, and were created by the people in the Loess Plateau region during the long-term struggle with soil erosion^25,26^. Check dams play an important role in reducing the amount of sediment entering the Yellow River, preventing floods and reducing disasters, and silting of fields, as well as consolidating the return of cultivated land to the forest (grass), ensuring ecological security, and promoting food production, the rational use of water resources, stable economic development, and stable social development^27,28^. The development of check dams has proceeded from small to large, from water storage and mud retention to silt production, and from single dam construction to the construction of dam systems. The history can be divided into four stages: the experimental demonstration stage began in the 1950s; the popularization stage occurred in the 1960s; the development and construction stage occurred in the 1970s; and the standardized construction stage began in the 1980s. As an important measure to control soil erosion, the number of check dams on the Loess Plateau has exceeded 50,000, with most distributed in the Shaanxi, Shanxi, and Inner Mongolia Provinces. However, the policy has changed in the twenty-first century, and the construction of check dams has gradually shifted to the implementation of the de-risking and strengthening of existing check dams. Check dams have been strengthened by adding, repairing, and altering spillways, repairing water-damaged dams, dealing with dam leaks and cracks, and so on. Moreover, the reinforcement of check dams has a long way to go due to insufficient funds and other factors.

**Figure 3:**
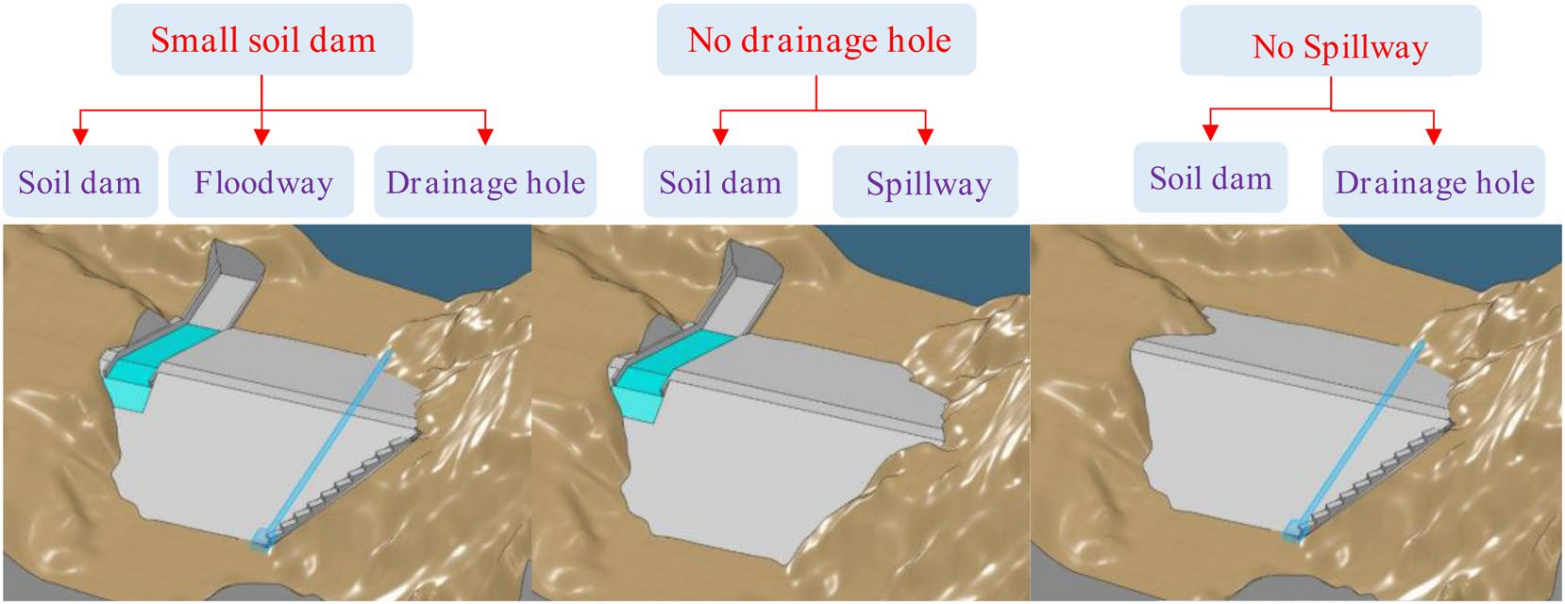
Structural types of traditional check dams.

According to the combined relationship of the main components of check dams, the structure of traditional check dams has three types, as shown in Fig. 3. However, some medium-sized check dams have only dam bodies and spillways and no drainage holes; in contrast, some check dams have only dam bodies and drainage holes and no spillways. There are different degrees of water damage and safety hazards. Some check dams are in regions in which special industries develop quickly, and the contradiction between the large amount of water used during the flood season and the inability of check dams to store water is becoming increasingly prominent. In addition, some check dams have been full of silt or have lost water storage function^29^.

Moreover, check dams with homogeneous soils, which break in the case of extreme floods and produce flood hazards downstream, have become a technical bottleneck limiting their development. A traditional check dam is set up with a flood storage capacity, and an additional slurry masonry or concrete spillway is required to ensure that the dam does not collapse under the standard flood design, but their cost is very high. Therefore, when a traditional check dam is designed, the inability of the dam to cross the flow has been a technical bottleneck, leading to three major problems: the high risk of collapse, high pressure on management and maintenance, and inadequate sand retention. These three problems have restricted the construction development of check dams, and the construction of check dams has been stopped in the last decade^30,31^.

#### High risk of collapse

The body of a check dam is filled with homogeneous soil, the dam body cannot overflow, and most of the check dams are not set up with spillways or they are set up with low defence standards. When a diffuse dam encounters a flood, the dam body very easily collapses, resulting in the siltation of stockpiled sediment, which is then released, and the danger caused by the release of uncoordinated water and sand is serious. For example, in July 2013, the Yan River in Shaanxi Province experienced continuous heavy rainfall, and 1309 check dams were damaged; in August 2016, the Darat Banner Xiliugou and Hantai River subbasins in the Inner Mongolia Autonomous Region experienced exceptionally heavy rainfall, causing severe water damage to the check dams, and 19 were damaged. Significant economic losses were caused by the resulting disasters.

#### High pressure on management and maintenance

The number of existing check dams in the Yellow River basin is approximately 58,800, and most check dams are distributed on the Loess Plateau area at all levels of branch ditches. The locations of the dams are scattered, and the risk of breakage due to flooding is high; these factors result in a high level of flood control. To effectively control the risk of disasters and ensure the safety of people's lives and property, relevant departments have repeatedly issued documents to strengthen the management and care of check dams and increase management and maintenance. The cost of human and financial resources for the management and maintenance of check dams has placed great pressure on the local government.

#### Inadequate sand interception

A traditional check dam is designed based on sediment siltation elevation, according to the standard flood level and considering the safety of water storage, and then the height of the dam is then determined. The retained storage capacity is set based on the standard flood level and to ensure dam safety. However, this retained storage capacity can be used only for flood control and not for sand interception, and the reservoir must be emptied to meet the flood capacity during the flood season. The statistical results show that the retained storage capacity of the existing check dams on the Loess Plateau area is approximately 40% of the total storage capacity, which means that approximately 2.24 billion m^3^ of the retained storage capacity cannot play a role in sand interception.

### Concept of the new check dam

In recent years, the development of check dam construction has attracted widespread attention. Many important instructions have been issued several times. The check dam is an effective measurement of the level of integrated management in a watershed^32^. The newly proposed the Ecological Protection and High Quality Development of the Yellow River Basin is a major national strategy, and check dams should be built vigorously in places where they can achieve the goals of soil and water conservation and environmental protection. The construction of check dams puts forward new requirements, such as high standards, free management and new technology. New check dams should be built in areas with intense soil erosion. The cross-regional check dam information monitoring mechanism should be constructed to achieve dynamic monitoring of and security risk warnings for important check dams. Additionally, new materials, technologies, and techniques should also be actively promoted and applied where the conditions of check dams are suitable.

**Figure 4:**
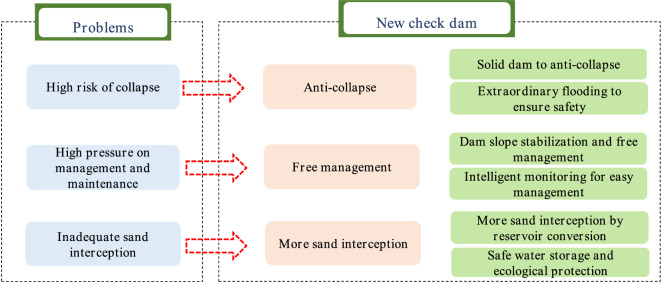
The concept of a new check dam.

There is a new check dam concept, which is shown in Fig. 4. The check dam should be constructed in areas with intense soil erosion. Small watersheds should be used as construction units, and large check dams should be used as control nodes to achieve a scientific layout of medium and small check dam systems. When adopting the concept of overall planning and scientific layout, the role of the dam system in sand control can be fully considered, and the overall flood control safety of the dam system can be considered in an integrated manner. First, new check dam is designed for anti-collapse characteristics. It should meet the requirements of the current specifications and be able to resist collapse. When there is an above-average flood, the dam should not collapse or slow collapse, and thus, the safety of people's lives and property can be ensured. Second, the check dam should have free management. The project should be safe and reliable. Supporting intelligent monitoring facilities and standardized management measures, the pressure of management and maintenance is further reduced. Third, the check dam should intercept more sand. Regarding safety, the project allows for as little unused reservoir capacity as possible, and more adequate and more significant benefits of sand interception can be obtained.

**Figure 5:**
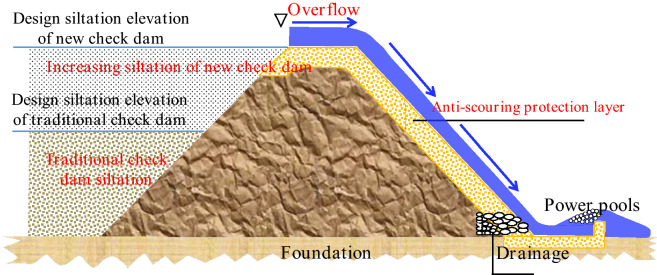
Schematic diagram of the new high-standard check dam.

In addition, with the gradual implementation of the rural revitalization strategy, the construction of check dams should have a water storage capacity that is able to improve production and life. Therefore, the new check dam should meet the requirements of the current specification under the precursor of reasonable planning and scientific layout and should completely consume normal flood waters and not collapse or slowly collapse during above-average floods. Moreover, the cost should be close to that of traditional check dams while considering into account water storage and ecological functions. Therefore, a comprehensive innovation of check dam construction materials, flood design, dam structure, and construction technology is carried out. The three problems of traditional check dams are solved by setting up an anti-scouring protection layer on the surface layer of the traditional check dam. A schematic diagram of the theoretical model is shown in Fig. 5.

### The key technology of the new check dam

Under the current situation of incongruous water–sand relationships, a new check dam system is proposed to solve the problem of dam overflow and enable the realization of ecological protection and restoration benefits. Based on new materials for solidified loess, new methods for hydrological calculations in small watersheds, new dam structures, and new technologies for design and construction, theoretical and technical systems for new check dams are proposed in this paper. The technical and economic benefits are compared with the traditional check dams and the new check dams can achieve the goals of anti-collapse, free management and maintenance, more sand interception, and cost reduction, thus better realizing the comprehensive benefits of check dams and supporting the Ecological Protection and High Quality Development of the Yellow River basin. The three innovative technologies of the new check dam design are described below.

### New material for solidified loess

The traditional anti-scouring protection layer is composed of sand and gravel aggregate concrete materials, but with the shortage of sand and gravel aggregates and the increase in cost, local material has become the preferred material for engineering construction in the Loess Plateau area. The use of locally sourced material can not only reduce the cost of the project but also reduce the construction period by using strong and durable solidified loess as a protective layer material against scouring. It has attracted the attention of many scholars and engineering industries. According to the classification of particle composition, loess is a medium-powder loam; generally, its clay particle content is more than 20%, and its powder particle content is less than 80%.

**Figure 6:**
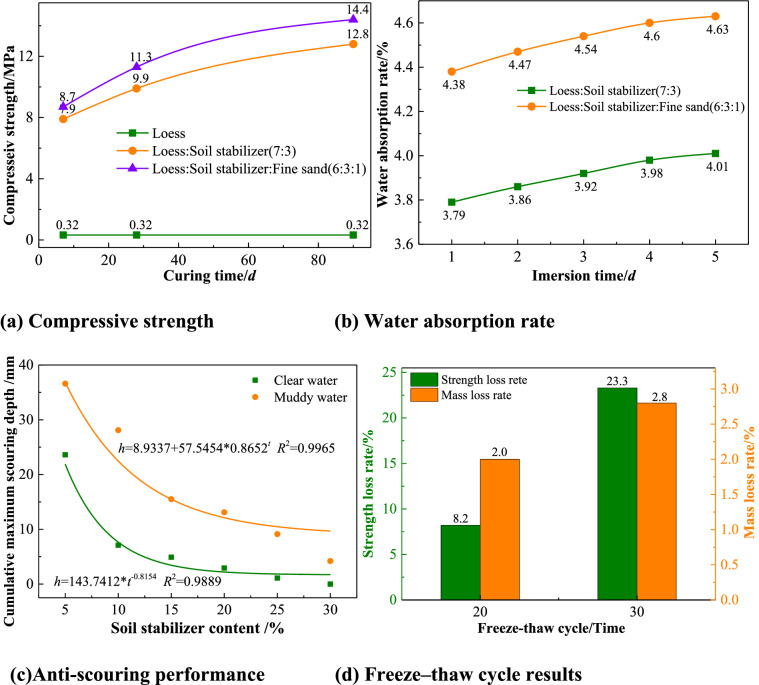
Results of new solidified loess.

In response to the technical problems of lack of sand and gravel materials on the Loess Plateau, as well as low strength and poor anti-scouring of traditional solidified soil, which cannot meet the overflow of the dam face, an anti-scour composite loess stabilizer was invented based on alkali-induced gelling soil solidification technology. It has high strength and good durability, providing a good solution to the problem in which traditional materials cannot meet the requirements of anti-souring. The test results are shown in Fig. 6. The 28-day compressive strength reached more than 9.9 MPa with 30% soil stabilizer content. The water absorption rate did not change significantly with increasing water immersion time and remained within 5%. The strength loss rate was 23.3% and the mass loss rate was 2.8% after 30 freeze–thaw cycles under a 25% soil stabilizer content. At a 15 m/s flow rate, the cumulative maximum scouring depth of solidified loess is less than 5 mm under a 30% soil stabilizer content. The solidified loess had good durability and anti-scouring characteristics and could meet the overflow and anti-scouring requirements of the new check dam design.

### New method of hydrological calculation

**Figure 7:**
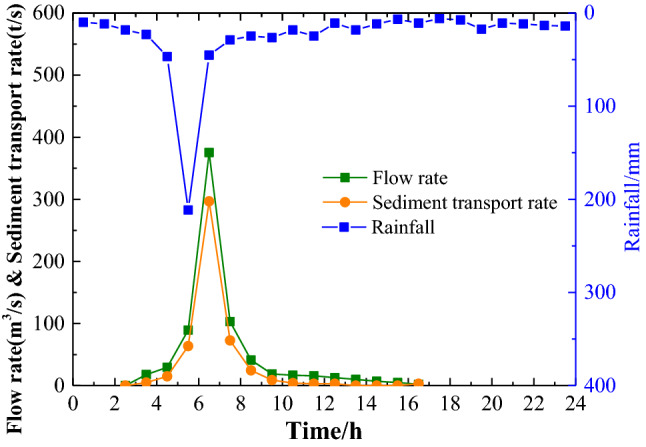
Water–sand relationship in the Loess Plateau area.

The fundamental reason for the complexity of the Yellow River is that there is less water and more sand, and the water–sand relationship is uncoordinated, which is a typical characteristic that distinguishes the Yellow River from other rivers, as shown in Fig. 7. To study this uncoordinated water–sand relationship problem more deeply, the author proposed the concept and calculation method of the coordinated degree of the water–sand relationship. The smaller the watershed area and the lower the river classification of the Loess Plateau branch ditch are, the lower the coordination of the water–sand relationship is, and the more check dams will need to play a role in the control area^33^. Therefore, the new check dam design stores poorly coordinated water and sand in situ and thus reduces the incompatibility of the water–sand relationship incompatibility from the source.

Generally, rainfall occurs randomly with equal probability at the basin-wide scale, but due to the differences in tributary basin areas, the probability of flooding in different tributaries (or subbasins) is spatially inconsistent, resulting a flooding probability of the same magnitude in larger basins under the same rainfall probability conditions, which are being much smaller than that in subbasins. According to the relevant specification^34^, the frequency method or empirical formula method was used to calculate the design flood of a check dam based on the actual measured data from larger watersheds or empirical formulae and atlases formulated by the actual measured data of larger watersheds. The basic assumption is that the probability of flood occurrence (frequency) of larger watersheds is the same as that in sub-basins. The check dams are in small watersheds that are prone to extreme local rainstorms. The traditional hydrological calculation method, i.e., the frequency method or empirical formula method, are used to calculate the design flood, and this method may lead to two consequences: the design flood frequency is low, or the design flood magnitude is small; thus, the check dam is objectively vulnerable to above-average floods and leads to dam collapse. Considering that special small watersheds are very prone to storm floods close to the upper limit of the boundary and that the body of a check dam can overflow without the need for flood storage capacity^35^, the maximum probable flood value is used to review the safety of the overflow of the dam body.

Currently, the maximum probable flood value is typically used for important water conservancy and hydropower projects and nuclear power plant projects^36,37^. However, the maximum probable flood value is not used in small watersheds^38^. Additionally, there is no precedent for calculating the maximum probable flood considering higher sand content. The new check dam design uses the probable maximum flood (PMF) to estimate flood sediment to cope with the extreme susceptibility of small watersheds close to the physical upper limit, and the dam is constructed with overflow structures without flood storage capacity. According to the Loess Plateau area characteristics and the law of flood and sand production, especially considering the influence of the 800 kg/m^3^ high sand content flood on the dam body scouring in the small watershed, the small watershed high sand content PMF estimation method for the new check dam hydrological calculation is proposed to solve the problem that the flood peaks in the special small basin are formed by short-term storms, the rainfall observation data are few and the extreme values are difficult to obtain. The method to derive the PMF flow is shown in Eq. (1). The new method of hydrological calculation fills the gap in the design of the possible maximum flood with high sand content in the special small watershed and establishes the upper limit of the flood sediment boundary for the anti-scouring protection layer of the check dam.

1 $$Q_{{{\text{PMF}}}} = 170.38 \cdot S^{0.6059}$$

where, $Q_{{{\text{PMF}}}}$ is small watershed high sand content PMF, (m^3^/s); $S$ is catchment area of small watershed, (km^2^).

### New technology of design and construction

Aiming at the technical bottleneck in which the traditional check dam is a homogeneous soil dam and the granular structure of the dam body cannot overflow, we propose setting up an anti-scour protection layer on the dam surface using the new materials of solidified loess so that the new check dam can discharge the flow through the dam body to achieve no collapse or slow collapse when encountering floods exceeding the standard, which essentially reduces the risk of dam collapse. Moreover, the retained storage capacity is fully utilized, which can not only provide adequate sand interception but also avoid scrapping and strengthening after siltation and reduce management and maintenance pressures. It will achieve a revolutionary breakthrough in the concept of the construction and renovation of check dams under extreme rainfall conditions in small watersheds.

The design and construction of the new check dams mainly address the anti-scouring protection layer. Three techniques are carried out according to the conditions of dam formation and anti-scouring protection requirements according to local conditions.

#### Full-coverage technology for the anti-scouring protective layer

**Figure 8:**
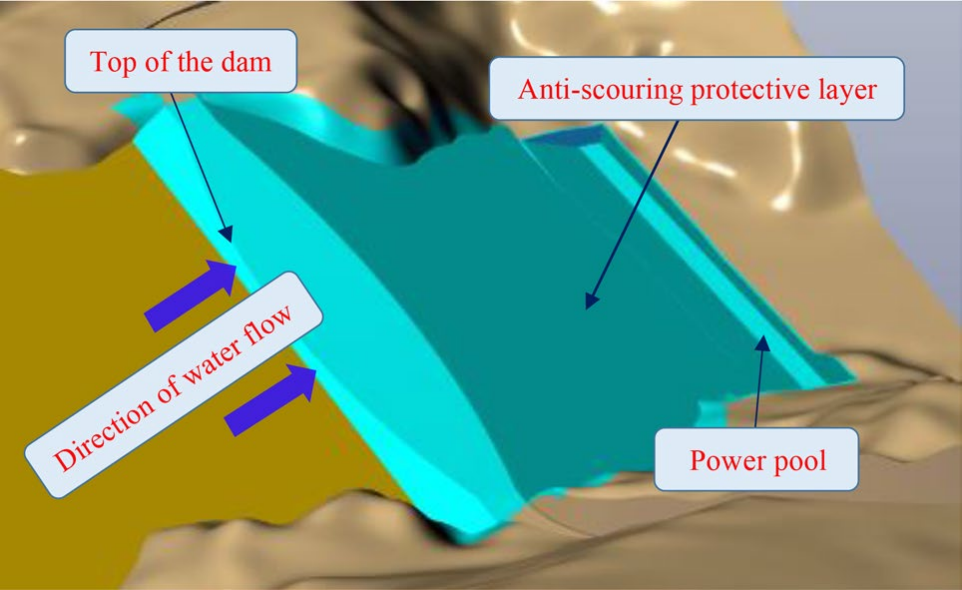
Full coverage technology for the anti-scouring protective layer.

When there are no sand and gravel aggregates in the check dam construction area, it is possible to use solidified loess as filling material, and the design of the top and downstream slope of the check dam uses the anti-scouring protection layer. This method is suitable for low and medium check dams where the height is below 20 m, as shown in Fig. 8. The flat rolling method is used to construct the anti-scouring protection layer and the dam body for the new check dam. The slope rolling method is used to transform the old check dams. The road mixing method, with high mechanization and fast construction speed, is used for mixing and paving.

#### Local coverage technology for the anti-scouring protective layer

**Figure 9:**
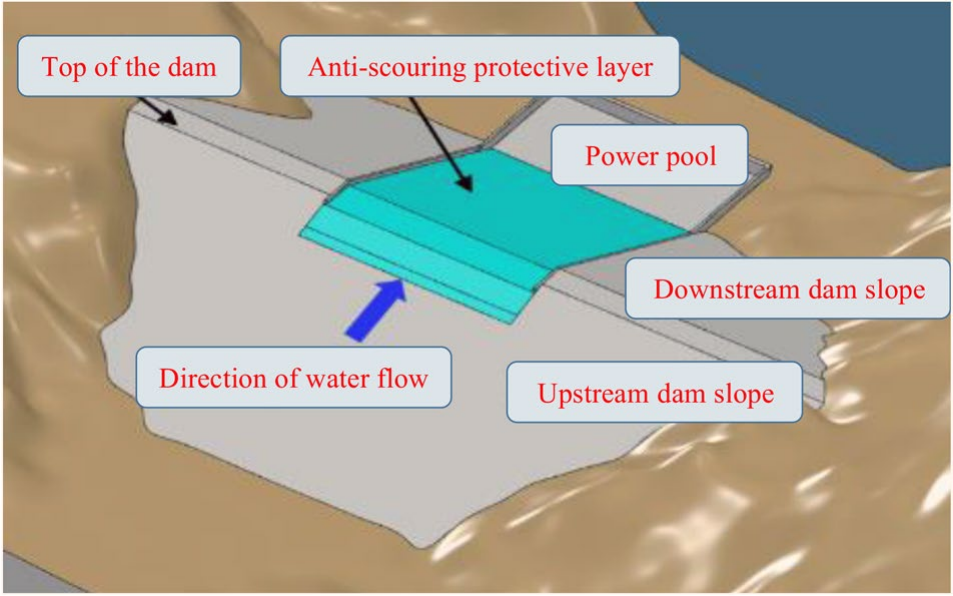
Local cover protection technology for the anti-scouring protective layer.

When the axis of the dam is long, the anti-scouring protective layer can be set up on the top and on the downstream slope of the dam, and the cost of the project can be reduced, which is suitable for the creation of a wider dam, as shown in Fig. 9. It is necessary to fill the dam body first to build a new dam and to excavate the overflow dam section to transform an old check dam. The slope rolling method is adopted to construct an anti-scouring protective layer for new check dams or overflow sections of old check dams.

#### Full-coverage technology for prefabricated interlocking blocks

**Figure 10:**
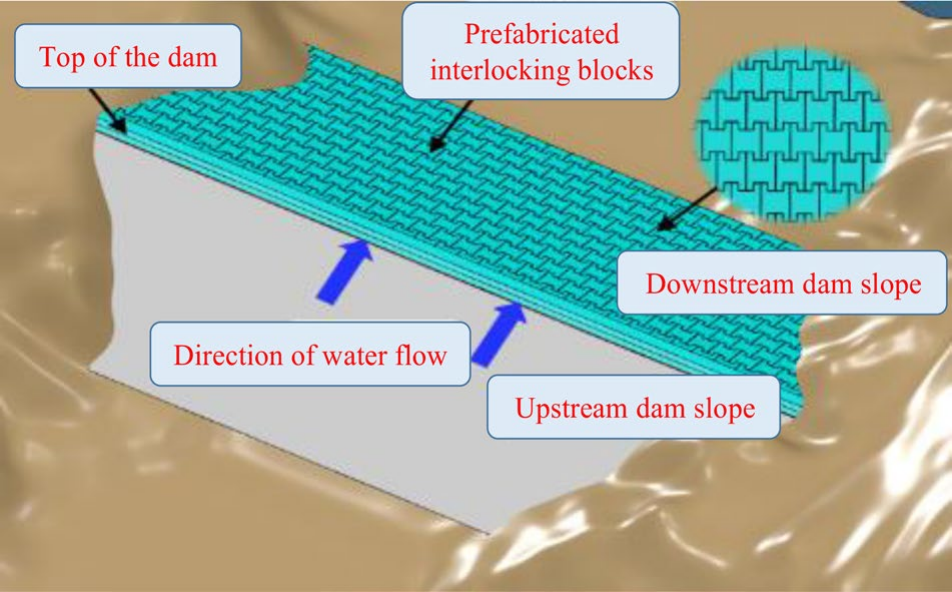
Full-coverage protection technology for prefabricated interlocking blocks.

For safety, the anti-scouring protection layer is adopted on the top of the dam, and prefabricated step-type interlocking blocks are used on the downstream slope as the anti-scouring protection layer; this method is used for dams with heights between 20 and 30 m, as shown in Fig. 10. During construction, the prefabricated interlocking blocks are made in a factory. The construction site conditions are low, the construction speed is fast and the quality is easy to control.

### Construction of an intelligent monitoring system for soil and water conservation

**Figure 11:**
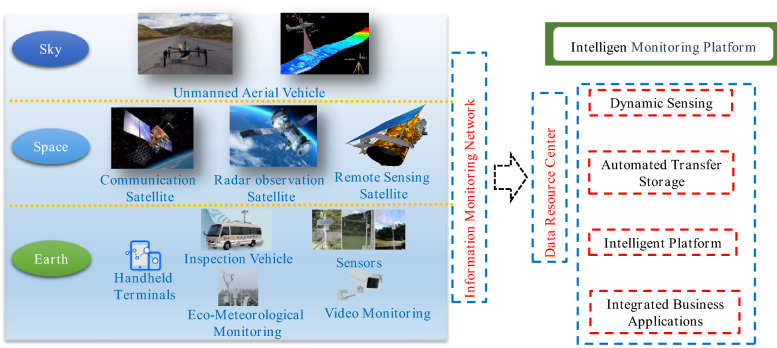
Framework of the intelligent monitoring system for soil and water conservation^43^.

Information technology was born in the last century and is now widely used in various fields because of its advantages in improving the efficiency of projects and reducing the consumption of human and material resources. Soil and water conservation monitoring improves soil and water conservation management; monitoring covers the whole management area, and the monitoring control system can achieve two types of functions^39,40^. One is for normal demand, which enables managers to quickly perceive changes in various types of conditions in the soil and water conservation area. The other is for extraordinary demand, which requires the timely perception of abnormal behaviour, such as soil and water conservation violations, and facilitates the timely handling of abnormal events. It accurately grasps the current situation of soil and water conservation covering the whole management and maintenance area and reflects the effect of soil and water conservation. It can realize the dynamic monitoring of soil erosion occurrence, development, hazards, and change trends and quickly perceives abnormal behaviour such as soil and water conservation violations. Then, security risk warnings can be issued. In turn, this approach can effectively improve the information management level of soil and water conservation in the Loess Plateau area and provide services for soil and water conservation protection and management development. The purpose is to build a soil and water conservation monitoring system that covers the entire check dam area to provide services for scientific research, production, and the management of soil and water conservation work on the Loess Plateau, thus effectively improving the level of soil and water conservation information. In turn, its functions can be realized: one is to quickly obtain all kinds of key data on soil and water conservation, with a big data analysis function, and the other is to detect abnormal behaviour of soil and water conservation quickly and issue security risk warnings as necessary^41,42^. It is imperative to build an integrated monitoring and early warning platform for check dams and develop new technologies to dynamically monitoring of check dam safety. The monitoring includes the basic situation of topography and geomorphology, vegetation, hydrometeorology, land use, soil and water conservation measures, quality, soil erosion degree, and soil erosion status. The framework of this design idea is shown in Fig. 11^43^, and the design integrates the use of satellite remote sensing, unmanned aerial vehicles, video intelligent monitoring, the ground Internet of Things, and other methods to build an efficient and collaborative integrated monitoring system of the sky, space and earth, forming a point, line, and surface space with full-coverage sensing capability, and big data analysis monitoring and processing can be conducted^44^.

### Information monitoring network

Different modules in the intelligent monitoring system play different roles, which ultimately constitute the information monitoring network. The details are described as follows: First, several solar-powered base stations are built in the Loess Plateau area, and the base stations are set up with automatic cruise-type UAVs to realize the whole process of automated intelligent inspection from UAV flight control, data acquisition, transmission, processing, and analysis, resulting in the formation. The UAVs are equipped with different types of sensors to obtain high-definition images and high-precision terrain data of key monitoring areas in real time; the actual situation of the site is captured for subsequent data analysis. Second, based on remote sensing images, thematic information such as land use distribution, vegetation coverage, soil distribution, and water conservation measures can be decoded and extracted to establish a database of soil and water conservation information and provide background data for the development of soil and water conservation work. Then, the time-series InSAR technology is used to monitor the surface deformation in real time by using the radar wavelength interference principle and to perceive the soil and water conservation engineering measures and slope stability from a macroscopic perspective in time, which has the advantages of providing a large monitoring range as well as all-weather, high accuracy and strong anti-interference ability characteristics. Finally, relying on the Internet of Things platform and 4G + Beidou communication technology, the distributed monitoring of soil temperature, moisture, rainfall, and other ecological environment quantities is investigated on the Loess Plateau. Combined with remote sensing data, a big data sensing layer of soil and water conservation is established on the Loess Plateau.

In summary, these research results show that the construction of the safety monitoring system of key check dams should be strengthened, and the construction of the information monitoring network of check dams should be realized based on the IoT acquisition platform and high-precision altitude sensors using the design concept of standardization and low cost; together, this approach can be used to achieve the high-precision observation of macro-deformation monitoring of dams, key parts and elements.

### Data resource centre

**Figure 12:**
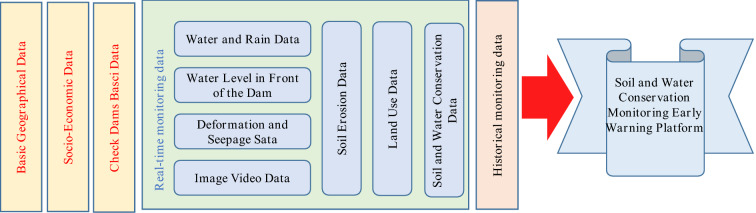
Construction model of the data resource centre.

The basic attributes, spatial geography, real-time monitoring, intelligent analysis, hydrometeorology and other check dam data are centrally stored, and the data are fused and efficiently managed to form a standard basic data resource, which provides the data basis for the development of the monitoring and early warning platform of check dams, as shown in Fig. 12. Establishing a data resource centre in the basin will enable the scientific management of data during the check dam monitoring period and ensure data security and the normal operation of the application system so that the application system can be quickly restored. By constructing the check dam database, the scattered information resources can be safely and scientifically managed to meet the data access needs of different nodes, thus providing services for the hierarchical management of soil and water conservation data, reporting at each level, and efficient sharing and exchange of information resources. Analytical studies can be carried out on topography and geomorphology, soil erosion status, land use status, check dam distribution, engineering construction dynamics, monitoring station distribution, water data, water level, sediment siltation and other data in the Loess Plateau area. This approach comprehends the construction and operation of soil and water conservation in a timely, accurate and comprehensive manner and provide services such as the querying and analysis of rain and water information.

### Intelligent monitoring and early warning platform

**Figure 13:**
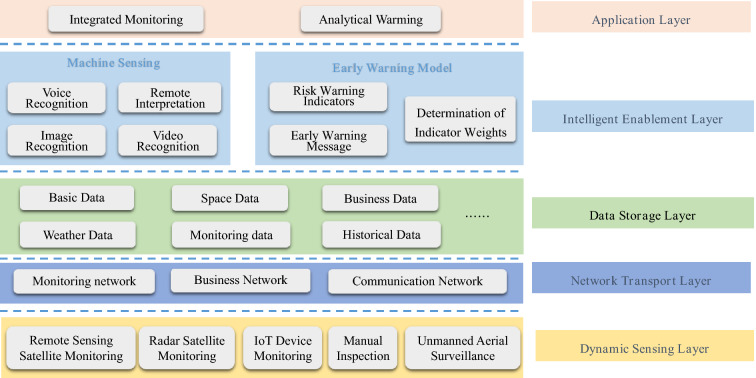
Framework of the intelligent monitoring and warning platform for soil and water conservation.

Based on the information monitoring network and the data resource centre, a monitoring and early warning platform with dynamic sensing, transmission and storage, an intelligent enabling layer, and a business application layer as the overall framework was built in this study, as shown in Fig. 13. A wisdom platform was built to unify the management of a learning algorithm library and a perception enabling library and provide intelligent analytical services such as prediction models and evaluation models for business applications through interface calls. The research results provide comprehensive technical support for dynamic monitoring, effect evaluation, and scientific decision-making in soil and water conservation areas. The sky-space-earth integration collection and monitoring data are integrated to mine, analyse, and research the current situation data and historical data. The results provide supervisory, management and decision support for soil and water conservation work. An intelligent interpretation system based on a deep learning algorithm is integrated to achieve the intelligent automatic identification of specific targets, comparative analysis, and supporting normalized fine monitoring and decision analysis of soil and water conservation. Video information is deeply mined, and artificial intelligence is used to carry out intelligent identification and classification. These new technologies can free up human resources while minimizing the amount of video communication information.

## Discussion

The soil and water conservation measures and projects currently implemented on the Loess Plateau have achieved significant ecological benefits, and the regional ecosystem services are developing in a healthy direction. With the advancement of science and technology and the development of the regional economy and society, more attention has been given to the integrated management model of small watersheds with both ditches and slopes. The soil and water conservation management projects on the Loess Plateau have significant holdings and play a controlling role in slowing down soil and water erosion, which must now shift from quantitative growth in soil and water conservation management to qualitative consolidation, enhancement and improvement.

Check dams are an effective measure to improve soil and water conservation on the Loess Plateau. The three major technical problems faced by traditional check dams (high risk of collapse, high pressure on management and maintenance, and inadequate sand interception) have affected their high-quality development. The proposal of a new check dam effectively solves the three major technical problems and provides a scientific basis for the construction and promotion of check dams with new materials, structures and technology. The study of traditional and new check dams was systematically carried out, and the results are shown in Table 1. The comparative analysis showed that the new check dams have better promotion prospects. The new check dam innovates the design and application concept, and builds a complete set of dam engineering design and construction technology. The PMF estimation method for small watersheds was developed, and a new hydrological calculation method for check dams was proposed, breaking through the PMF estimation technology for small watersheds with high sand content on the Loess Plateau. A new material for loess solidification has been developed with the soil stabilizer, which has high strength and good durability, and can be used as the filling material for the new check dam. Technological innovation has been comprehensively carried out in dam engineering design and construction as well as hydrological calculations and new materials.

**Table 1:** Comparison of design concepts and ideas for check dams.

Traditional check dam	New check dam
The dam is not overflowable and has a high risk of collapse	The dam can withstand standard floods and above-standard floods and prevent breaching
Dam slopes are vulnerable to damage, heavy flood control tasks, and high pressure on management and maintenance	The scour protection layer has high strength, low water absorption rate, and good durability, which can achieve the goal of being maintenance-free
Retained storage capacity can guarantee dam safety, but sand retention is not sufficient	Retained storage capacity can be partially converted into sand control capacity, which can offer more sand control

With the help of modern information technologies such as big data mining, geospatial analysis, and geoscience information maps, the valuable experience of soil and water conservation and governance on the Loess Plateau was deeply excavated and new knowledge and new rules were formed. In the future, it is necessary to closely focus on the reform and development goals of soil and water conservation, and establish an integrated air-ground supervision platform. It highlights the macro-analysis characteristics of remote sensing data, comprehensively improves the modernization level and efficiency of soil and water conservation supervision, and accelerates the application of new technologies and methods of soil and water conservation supervision. The new model will provide strong support to prevent soil erosion and promote the construction of an ecological civilization. Finally, the ambitious goal of promoting the construction of intelligent soil and water conservation with digital twin watersheds will be achieved.

## Conclusion

Soil erosion is serious, and the ecological environment is fragile in the Loess Plateau region. Engineering, biological measures, and farming measures are all necessary to inhibit soil erosion in the Loess Plateau region, reduce the amount of sediment entering the Yellow River and improve the ecological environment. For the three major problems inherent in traditional check dams, such as the high risk of collapse, high pressure of management and protection, and insufficient sand retention, the concept of a new high-standard check dam was redefined in this paper. Thus, key technology research, such as new materials for loess solidification, new methods for hydrological calculation, and new technologies for the design and construction of new check dams, was carried out to achieve the identified goals of check dams, such as breach prevention, safety, free management, more sand interception, and higher standards. The comprehensive benefits of check dams are better utilized, which in turn supports the Ecological Protection and High Quality Development of the Yellow River Basin. A unified and stable sky-space-earth integrated intelligent monitoring system for soil and water conservation was built by monitoring technologies such as satellite remote sensing, unmanned aerial vehicles, remote video monitoring, automatic observation, and the internet. It is important for the timely and accurate acquisition and feedback of dynamic information on soil and water conservation construction and management in the Loess Plateau region, strengthening the technical guidance available for soil and water conservation and improving the scientific and objective evaluation of soil and water conservation on the Loess Plateau. The research results provide new ideas, methods, and technologies for realizing soil and water conservation in the Loess Plateau region.

## Data Availability

The data that supports the findings of this study are available upon request from the corresponding author. The data are not publicly available due to privacy or ethical restrictions.
